# The Prevalence of Diamine Oxidase Polymorphisms and Their Association with Histamine Intolerance Symptomatology in the Mexican Population

**DOI:** 10.3390/biomedicines13092280

**Published:** 2025-09-17

**Authors:** Pamela Aguilar-Rodea, Viviana Mejía-Ramírez, Raúl Hernández-Munguía, Saúl Ramírez-Vargas, Diana Tovar-Vivar, Jaquelin Leyva-Hernández, Juan Carlos Nacar-Gutiérrez, Miriam Morales-Martínez, Aracely Palafox-Zaldivar

**Affiliations:** Laboratorios Columbia S.A. de C.V., Panzacola 62, Villa Coyoacán, Coyoacán, Mexico City 04000, Mexico

**Keywords:** diamine oxidase, DAO deficiency, polymorphisms, SNP inheritance, histamine intolerance

## Abstract

Exogenous histamine obtained from the intake of histamine-rich food is mainly metabolized by the diamine oxidase enzyme (DAO). Histamine intolerance (HIT) is an alteration mainly caused by DAO deficiency, which is commonly associated with gastrointestinal, respiratory, cardiovascular, central nervous system, muscular, skeletal, and skin symptoms. Despite four single-nucleotide polymorphisms (SNPs) being mainly associated with DAO deficiency, the probability of inheriting these variants and their relationship with HIT in the Mexican population remain unknown. **Objective:** The aim of this study was to evaluate the prevalence of these SNPs and their relationships with HIT in the Mexican population, including both individual volunteers and family groups. **Methods:** Four SNPs related to DAO deficiency were detected in 112 volunteers; medical questionnaires were answered. **Results:** The prevalence of genetic DAO deficiency attributed to at least one risk allele was 78.57% (rs1049793 was the main SNP). Fifteen DAO SNP combinations were detected (the main rs2052129, rs10156191, rs1049742 (wild-type homozygotes), and rs1049793 (heterozygote), 31.25%). A total of 41.07% of the volunteers presented at least three symptoms in different systems related to HIT, of whom 84.78% presented at least one SNP. The DAO deficiency genetic risk score varied among individual volunteers and families. The highest probability of having a mutated homozygote was 11.8% (rs1049793). HIT symptoms varied among relatives sharing identical genotypes. **Conclusions:** The prevalence of SNPs related to DAO deficiency in the Mexican population correlates with globally reported data; however, further analysis with volunteers distributed throughout the country would be desirable. Although genetic predisposition was common, the presence of SNPs alone did not predict specific HIT symptoms. Multiple SNPs may increase the presence of HIT symptoms, regardless of the type of allele. These findings highlight the multifactorial nature of HIT and underscore the need for standardized diagnostic criteria.

## 1. Introduction

Histamine is an amine of endogenous (from histidine) and exogenous (from the intake of histamine-rich food) origin which is highly distributed in the human body. It is synthesized and stored in mast cells, basophils, platelets, histaminergic neurons, and enterochromaffin cells and is responsible for several functions in the body—including in the immune, digestive, and nervous systems—by binding to four receptors (H1, H2, H3, and H4) distributed in different tissues [1,2,3,4].

Two enzymes are responsible for histamine metabolization depending on its location:

(1) diamine oxidase (DAO), due to oxidative deamination in the case of extracellular histamines, and
(2) histamine-N-methyltransferase (HNMT), via cyclomethylation for intracellular histamines [4,5].

DAO is a secretory enzyme located in the upper intestinal mucosa chromaffin cells, principally distributed in the intestines, kidneys, placenta, and other organs. This enzyme is secreted into the gastrointestinal tract in response to a high concentration of histamine in the body, as its main function is to metabolize extracellular histamine obtained from food such as cheese, fish, processed meat, fermented meal, or alcoholic beverages [4,5,6].

Histamine intolerance (HIT) is described as an alteration in histamine homeostasis, mainly caused by decreased intestinal metabolization of exogenous histamines attributed to DAO deficiency [4,7]. HIT leads to the accumulation of histamines, mainly in the gut, producing adverse reactions [4,8]. Some of the clinical manifestations associated with HIT may include gastrointestinal, respiratory, cardiovascular, central nervous system, muscular, skeletal, and skin disorders [3,9,10], with recent studies also reporting a complex combination of these symptoms, which have been associated with the distribution of the four histamine receptors [1,10].

Regarding the most frequent and severe symptoms reported in patients with HIT, gastrointestinal manifestations—principally, abdominal distention, postprandial fullness, diarrhea, abdominal pain, irritable bowel syndrome, and constipation—are the most outstanding [1,11,12]. However, dizziness, headache, migraine, palpitations, fibromyalgia, muscle pain, fatigue, asthma, nasal congestion, dermatologic symptoms (eczemas), and Attention-Deficit Hyperactivity Disorder (ADHD) are also typical symptoms of interest in patients with HIT [12,13].

Three factors have been reported as the cause of DAO deficiency: (1) genetic alterations, (2) damage in the bowel mucosa due to inflammatory pathologies, and (3) inhibition of the enzyme produced by drugs [4,14,15]. However, a recent study conducted by Okutan and collaborators (2023) [3] showed that 74.5% of DAO deficiency cases can be mainly attributed to genetic factors.

DAO is encoded by the *AOC1* gene, which is located on chromosome 7 (7q34-q36) of the human genome. To date, 85 SNPs (single-nucleotide polymorphisms) have been associated with DAO deficiency; however, four SNPs stand out for being mainly associated with changes in the enzymatic activity of DAO in the Caucasian population: rs1049793 (His645Asp), rs10156191 (Thr16Met), rs2052129 (G4586T), and rs1049742 (Ser332Phe) [3,16].

Unlike point mutations, SNPs have been highly evolutionarily successful, being fixed mutations in at least 1% of the population [17]. SNPs can produce important changes in protein functions, being responsible for diversity between individuals and genome evolution, and are frequently related to specific phenotypes such as same-family features and particular diseases [18]. Certain SNPs are consistently inherited across generations and have been extensively studied through Genome-Wide Association Studies (GWASs) to identify potential associations—either positive (predisposition) or negative (protective)—between specific genotypes and various diseases. Particularly, in the case of the four DAO SNPs described above, a large amount of information has been reported; however, no studies related to the inheritance and maintenance of these SNPs in close family members and their association with clinical manifestations associated with HIT have been reported.

Given the limited information in our country, the aim of this study was to provide relevant data regarding the prevalence and inheritance of the four SNPs mostly commonly reported to be associated with DAO deficiency worldwide, as well as the various HIT-related symptoms these SNPs may cause in the Mexican population.

## 2. Materials and Methods

### 2.1. Study Design and Participants

An observational, descriptive cross-sectional study was conducted to analyze the prevalence and associations between four SNPs of the histamine-degrading enzyme DAO and the development of clinical manifestations related to HIT—mainly affecting the nervous system, gastrointestinal tract, skin, respiratory system, genitourinary system, and circulatory system, among others—in the Mexican population. For this study, a total of 112 volunteers were included: 53 volunteers grouped into families, including members of the same family (parents, children, brothers, grandparents, and uncles), with each family made up of at least two members, and 59 individual volunteers.

The Research and Ethics Committees from the Hospital La Misión, Monterrey, Nuevo León, México, (17 CI 19 039 096 and CONBIÉTICA-19-CEI-008-20160729) evaluated and approved the study protocol and informed consent (LC-DAO-SIH-2024-02 v 1.0), which involved a single non-invasive oral mucosa sample and a routine medical questionnaire. This study did not involve any medical intervention in the volunteers. The protocol was classified as risk-free research for the studied volunteers and was performed in compliance with the guidelines laid out in the Declaration of Helsinki and with the Good Clinical Practices (GCP) defined in the ICH E6 (R2). The volunteers were informed in detail about the aim and procedure of this study, and their agreement was obtained by signing a written informed consent form prior to inclusion; for children under 12 years old, their parents or legal guardians provided informed consent for the children’s inclusion.

### 2.2. Population and Biological Samples

A total of 112 volunteers of both genders, thirteen family groups (*n* = 53) and individual volunteers (*n* = 59), were recruited. Once informed consent was obtained, their medical history was recorded, which included hereditary family history and personal pathological background, including nervous, gastrointestinal, dermatological, respiratory, genitourinary, and cardiovascular system symptoms and others such as insomnia, Attention-Deficit Hyperactivity Disorder (ADHD), and fibromyalgia.

Each volunteer was given important instructions to ensure proper sample collection, including abstaining from eating, smoking, drinking, brushing their teeth, or using mouthwash for at least one hour prior to sampling.

A single non-invasive oral mucosa sample was collected by rubbing the inner side of both cheeks with two flocked swabs under sterile conditions, as described in Ramírez-Vargas’ thesis work [19]. Each swab was then placed into a separate sterile tube. The biological samples were stored at room temperature until processing.

### 2.3. Diamine Oxidase SNP Genotyping Analysis

Genomic DNA was extracted from buccal swabs using the salting-out method. Briefly, the two swabs were reconstituted in TBE 1x buffer. After vortexing and centrifugation, the supernatant was discarded (these steps were performed twice). Then, 400 µL of lysis solution, 25 µL of SDS 20%, and 9 µL of proteinase K were added. The samples were incubated overnight at 56 °C. Subsequently, 25 µL of saturated sodium chloride was added, and after centrifugation, the supernatant was transferred to a new tube. DNA was precipitated by adding 400 µL of absolute ethanol, mixing the solution, and incubating it at 4 °C for 10 min. Following centrifugation, the supernatant was discarded, and the DNA pellet was washed with 70% ethanol. After a final centrifugation step, the remaining ethanol was completely removed, and the DNA was rehydrated in 50 µL of nuclease-free water. DNA purity and concentration were assessed in triplicate using a Nanodrop One C spectrophotometer (Thermo Fisher Scientific, CDMX, Ciudad de México, Mexico). High-quality DNA samples were stored at −20 °C until further use.

Genotyping of diamine oxidase (DAO) single-nucleotide polymorphisms (SNPs) was performed using predesigned TaqMan^®^ assays (Applied Biosystems^®^, Thermo Fisher Scientific, Dreieich, Germany). Four diamine oxidase SNPs were included: rs2052129 (C_11630976_1), rs10156191 (C_25593951_10), rs1049742 (C_7599782_20), and rs1049793 (C_7599774_10) (see Table 1). Detection of the SNPs was carried out using a CFX96 and a CFX OPUS 96 Real-time PCR thermocycler System (Bio-Rad Laboratories Inc., Berkeley, CA, USA). Four independent reactions were performed (one reaction for each SNP) using the fluorescent reporter dyes VIC (wild-type genotype) and FAM (mutated genotype). The amplification conditions were as follows: rs2052129—initial denaturalization for 10 min at 95 °C, followed by 50 cycles (92 °C for 15 s and 58 °C for 1 min) and a final cycle of 25 °C for 1 min; rs10156191 and rs1049793—10 min at 95 °C, followed by 40 cycles (92 °C for 15 s and 60 °C for 1 min) and a final cycle of 25 °C for 1 min; and, finally, rs1049742—10 min at 95 °C, followed by 40 cycles (92 °C for 15 s and 65 °C for 1 min) and a final cycle of 25 °C for 1 min. Fluorescence signals for all analyzed SNPs were successfully measured at the end of each cycle and at the endpoint. Reactions were performed in triplicate. Genotypes were assigned based on the amplification curves and the allelic discrimination plots generated using the real-time PCR thermocycler system. The standardization of this methodology was described in Mejía-Ramírez’s thesis work [20].

### 2.4. Statistical Analysis

The genotype–phenotype relationships were assessed through mixed-effect, ordered logistic regression models, since the outcome variable was categorized as an ordered response based on the number of symptoms: “none,” “just one,” or “more than one.” This kind of model analyzes the clustered nature of data since some individuals were grouped into families, so the regression estimated both fixed and random effects. The regressors included were the SNPs categorized as binary predictors, with 0 for wild-type homozygotes and 1 otherwise, controlling for the age and sex of individuals. All procedures were performed with the statistical software Stata version 18.0.

Data distribution was performed using GraphPad Prism, version 9.4.1 for Windows, GraphPad Software, Boston, Massachusetts USA, www.graphpad.com [21].

The DAO deficiency genetic risk score previously described by Okutan and collaborators (2023) [3] was calculated to evaluate the cumulative presence of multiple DAO SNPs, which basically consist of the sum of all risk alleles (0 = wild-type homozygotes; 1 = heterozygotes; and 2 = mutated homozygotes). In addition, the impact of each SNP on DAO activity was considered based on the locus, frequency, and serum functional activity previously reported by other authors [16,22] (0 = rs1049742 and rs10156191, considered as natural variants; 1 = rs1049793, most frequent SNP, dysfunctional DAO activity; and 2 = rs2052129, promoter region, decreased transcriptional activity).

The z-test for the difference between two proportions was used to compare the frequency of the altered allele in our sample with that of the reference population, using the Stata command “prtesti”.

The probability of having different genotypes in the four analyzed DAO SNPs was determined under the Hardy–Weinberg equilibrium. Several chi-squared tests were conducted to assess whether the populations significantly deviated from Hardy–Weinberg equilibrium proportions.

## 3. Results

### 3.1. Study Population

This study was conducted between July and December 2024, including 112 volunteers of both genders: 13 family groups (*n* = 53) and individual volunteers (*n* = 59), comprising 55 females (49.11%) and 57 males (50.89%). Participants ranged in age from 7 to 85 years. A total of 13 families were included to assess the prevalence of DAO polymorphisms within the same family and their potential association with a common clinical manifestation of HIT. Participants were grouped according to kinship; three generations were studied in three families, two generations in nine families, and one generation in a single family (sisters).

**Figure 1:**
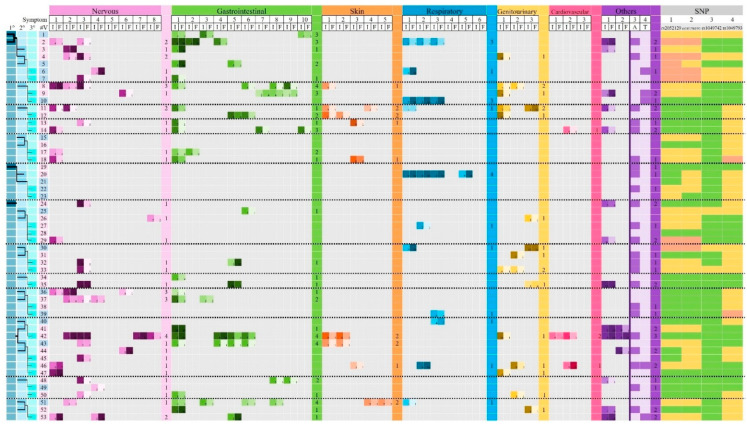
General data, pathological background, and DAO SNPs identified in volunteers grouped into families. 1°: first generation; 2°: second generation; 3°: third generation. Highlighted squares indicate the generation of each volunteer and, the lines, their genealogy. Dot lines separate each family. #V: number of volunteers; in pink, female; in blue, males. I: intensity (scale 1–10); F: monthly frequency: 1: 1–3 days; 2: 4–6 days; 3: 7–9 days; 4: 10–12 days; 5: 13–15 days; 6: 16–18 days; 7: 19–21 days; 8: 22–24 days; 9: 25–27 days; 10: 28–30 days. Symptoms: nervous system: 1: anxiety; 2: chronic fatigue; 3: headache; 4: nervousness; 5: panic attacks; 6: depression; 7: generalized weakness; 8: dizziness; gastrointestinal system: 1: bloating; 2: postprandial plenitude; 3: diarrhea; 4: abdominal pain; 5: constipation; 6: intestinal colic; 7: belching; 8: nausea; 9: vomiting; 10: flatulence; dermatological system: 1: pruritus; 2: redness; 3: eczema; 4: swollen eyelids; 5: red eyelids; respiratory system: 1: rhinorrhea; 2: rhinitis; 3: nasal congestion; 4: asthma/bronchospasm; 5: frequent sneezing; 6: dyspnea; genitourinary system: 1: menstrual cramping; 2: dysmenorrhea; 3: urinary symptoms; cardiovascular system: 1: hypotonia; 2: palpitations; 3: loss of consciousness; others: 1: insomnia; 2: fibromyalgia; 3: allergies; 4: ADHD (Attention-Deficit Hyperactivity Disorder). In the case of allergies and ADHD, only the presence of the symptom is highlighted. The total number of symptoms of each system is shown for each volunteer in the colored files. DAO SNPs rs2052129 (1), rs10156191 (2), rs1049742 (3), and rs1049793 (4) are also shown. Green: wild-type homozygotes; yellow: heterozygotes; red: mutated homozygotes.

**Figure 2:**
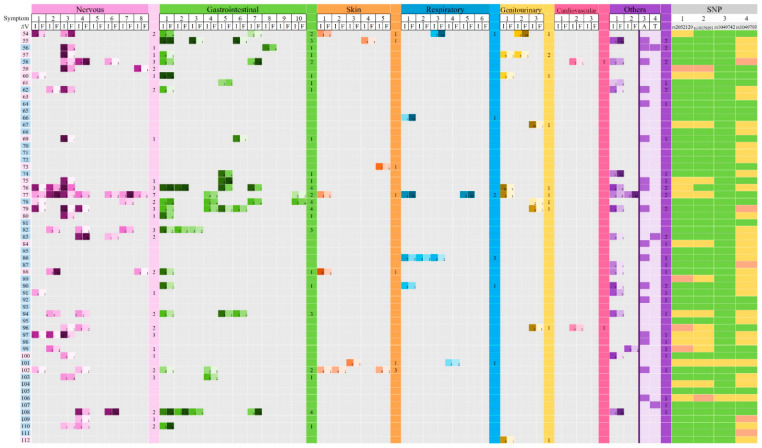
General data, pathological background, and DAO SNPs identified in individual volunteers. #V: number of volunteers; in pink, female; in blue, males. I: intensity (scale 1–10); F: monthly frequency: 1: 1–3 days; 2: 4–6 days; 3: 7–9 days; 4: 10–12 days; 5: 13–15 days; 6: 16–18 days; 7: 19–21 days; 8: 22–24 days; 9: 25–27 days; 10: 28–30 days. Symptoms: nervous system: 1: anxiety; 2: chronic fatigue; 3: headache; 4: nervousness; 5: panic attacks; 6: depression; 7: generalized weakness; 8: dizziness; gastrointestinal system: 1: bloating; 2: postprandial plenitude; 3: diarrhea; 4: abdominal pain; 5: constipation; 6: intestinal colic; 7: belching; 8: nausea; 9: vomiting; 10: flatulence; dermatological system: 1: pruritus; 2: redness; 3: eczema; 4: swollen eyelids; 5: red eyelids; respiratory system: 1: rhinorrhea; 2: rhinitis; 3: nasal congestion; 4: asthma/bronchospasm; 5: frequent sneezing; 6: dyspnea; genitourinary system: 1: menstrual cramping; 2: dysmenorrhea; 3: urinary symptoms; cardiovascular system: 1: hypotonia; 2: palpitations; 3: loss of consciousness; others: 1: insomnia; 2: fibromyalgia; 3: allergies; 4: ADHD (Attention-Deficit Hyperactivity Disorder). In the case of allergies and ADHD, only the presence of the symptom is highlighted. The total number of symptoms of each system is shown for each volunteer in the colored files. DAO SNPs rs2052129 (1), rs10156191 (2), rs1049742 (3), and rs1049793 (4) are also shown. Green: wild-type homozygotes; yellow: heterozygotes; red: mutated homozygotes.

Figure 1 and Figure 2 show data obtained from medical histories, particularly personal pathological backgrounds, including neurological, gastrointestinal, dermatological, respiratory, genitourinary, cardiovascular, and other conditions potentially associated with DAO deficiency. A total of 39 symptoms or clinical manifestations were evaluated, considering both the intensity and frequency of each symptom.

### 3.2. DAO SNP Genotyping

For each DAO SNP analyzed—rs2052129, rs10156191, rs1049742, and rs1049793—three possible genotypes were identified: (1) wild-type homozygotes, associated with normal DAO activity; (2) heterozygotes, associated with reduced DAO activity due to a partially defective enzyme; and (3) mutated homozygotes, corresponding to absent DAO activity caused by a fully non-functional enzyme, as described by Blasco-Fontecilla and collaborators (2024) (Figure 1 and Figure 2). 

**Figure 3:**
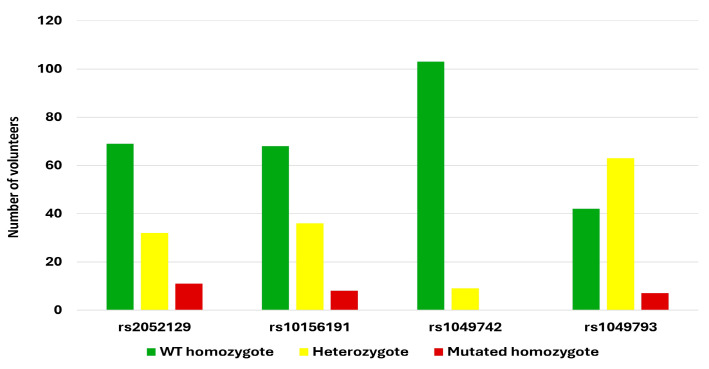
DAO SNP prevalence. DAO SNPs: 1: rs2052129; 2: rs10156191; 3: rs1049742; 4: rs1049793; green: wild-type homozygotes; yellow: heterozygotes; red: mutated homozygotes.

Among the 112 volunteers, the prevalence of genetic DAO deficiency—defined as the presence of at least one risk allele—was 78.57% (*n* = 88/112). The most frequently detected SNP carrying at least one risk allele was rs1049793 (62.5%; *n* = 70/112), followed by rs10156191 (39.29%; *n* = 44/112), rs2052129 (38.39%; *n* = 43/112), and rs1049742 (8.04%; *n* = 9/112). Across the four DAO SNPs analyzed, the distribution of genotypes was as follows: 62.95% wild-type homozygotes, 31.25% heterozygotes, and 5.80% mutated homozygotes. Notably, no mutated homozygotes were detected for the SNP rs1049742 (Figure 3).

**Figure 4:**
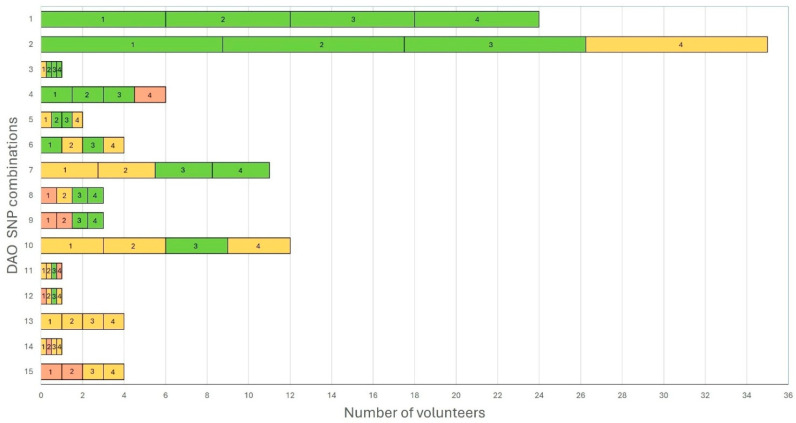
DAO SNP combinations (genotype combinations) and their prevalence. DAO SNPs: 1: rs2052129; 2: rs10156191; 3: rs1049742; 4: rs1049793; green: wild-type homozygotes; yellow: heterozygotes; red: mutated homozygotes.

A total of fifteen different DAO SNP combinations (genotype combinations) were identified among the volunteers. The most frequent was genotype combination 2 (rs2052129, rs10156191, rs1049742: wild-type homozygotes; rs1049793: heterozygote, *n* = 35), followed by genotype combination 1 (rs2052129, rs10156191, rs1049742, rs1049793: wild-type homozygotes, *n* = 24) and genotype combination 10 (rs2052129, rs10156191, rs1049793: heterozygotes; rs1049742: wild-type homozygote, *n* = 12) (Figure 4).

**Figure 5:**
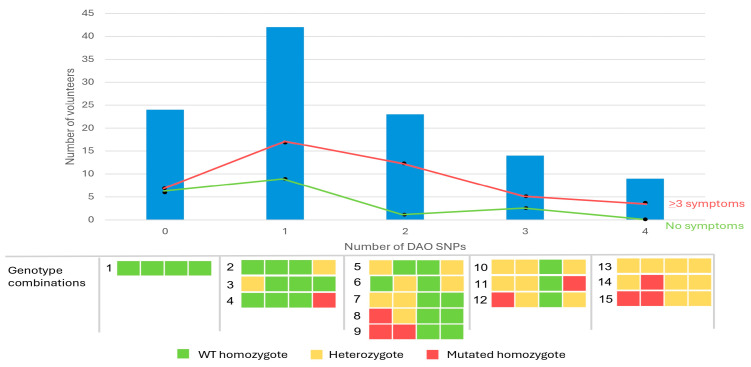
Number of DAO SNPs per volunteer and their relationship with HIT symptoms. WT: Wild-type. Genotype combinations: 1–15: DAO SNPs combinations (genotype combinations); first square: rs2052129; second square: rs10156191; third square: rs1049742; fourth square: rs1049793. Red line: Number of volunteers with at least three symptoms in different systems related to HIT; green line: number of volunteers without symptoms related to HIT. Total included symptoms: 39.

The number of DAO SNPs per volunteer and their relationship with symptoms related to HIT are shown in Figure 5: no DAO SNPs were found (genotype combination 1) in 24 volunteers (21.43%). A total of 42 volunteers (37.5%) carried one DAO SNP (genotype combinations 2 to 4); 23 volunteers (20.54%) carried two DAO SNPs (genotype combinations 5 to 9); 14 volunteers (12.5%) carried three DAO SNPs (genotype combinations 10 to 12); and finally, 9 volunteers (8.04%) carried four DAO SNPs (genotype combinations 13 to 15).

Among the volunteers presenting at least three symptoms related to HIT in different systems, those carrying only one DAO SNP were the most frequently identified (36.96%), followed by volunteers carrying two DAO SNPs (28.26%). All volunteers carrying four DAO SNPs presented symptoms (Figure 5).

### 3.3. Association Between DAO SNPs and Clinical Manifestations Related to HIT

**Figure 6:**
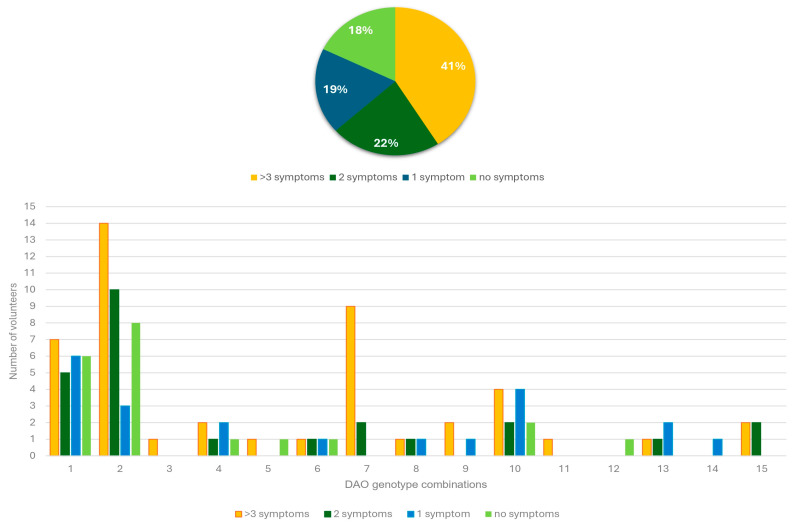
Number of symptoms in different systems related to HIT, reported by each volunteer, and their association with DAO genotype combinations.

A total of 46 volunteers (41.07%) showed at least three symptoms in different systems related to HIT (potential volunteers with HIT), with an average of 5 out of 39 symptoms per volunteer. One volunteer presented 16 symptoms. Among these volunteers, 84.78% presented at least one SNP. Genotype combination 2 was the most frequent (30.43%), followed by genotype combination 7 (19.57%) and genotype combination 1 (15.22%). Between the volunteers with less than three symptoms in different systems (healthy volunteers), with an average of one symptom per volunteer, the presence of at least one SNP was 74.24%, with genotype combination 2 as the main combination (31.82%), followed by genotype combination 1 (25.76%) and genotype combination 10 (12.12%); 20 volunteers with different genotype combinations did not report any symptom (Figure 4, Figure 5 and Figure 6 and Table S1). No difference between potential volunteers with HIT and volunteers considered healthy was observed in terms of genotype combination.

**Figure 7:**
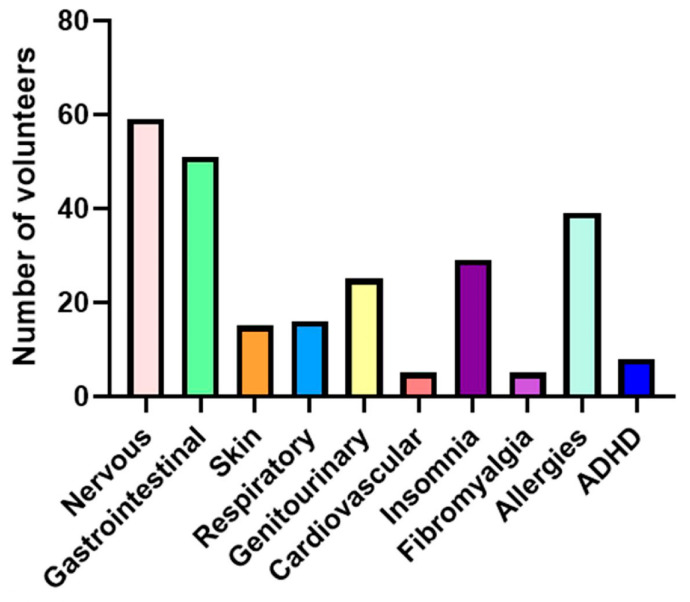
Number of volunteers with symptoms related to HIT. Symptoms included in each system: Nervous system: anxiety, chronic fatigue, headache, nervousness, panic attacks, depression, generalized weakness, dizziness; Gastrointestinal system: bloating, postprandial plenitude, diarrhea, abdominal pain, constipation, intestinal colic, belching, nausea, vomiting, flatulence; Dermatological system: pruritus, redness, eczema, swollen eyelids, red eyelids; Respiratory system: rhinorrhea, rhinitis, nasal congestion, asthma/bronchospasm, frequent sneezing, dyspnea; Genitourinary system: menstrual cramping, dysmenorrhea, urinary symptoms; Cardiovascular system: hypotonia, palpitations, loss of consciousness; Others: insomnia; fibromyalgia; allergies; ADHD (Attention-Deficit Hyperactivity Disorder).

The most frequently reported symptom was allergy (*n* = 39/112; 34.82%), followed by bloating (*n* = 37/112; 33.04%) and headache (*n* = 32/112; 28.57%) (Figure 1 and Figure 2). Symptoms related to the nervous system were the most prevalent, reported by 52.68% (*n* = 59/112) of the volunteers, followed by gastrointestinal symptoms (45.54%; *n* = 51/112) and allergic manifestations (34.82%; *n* = 39/112) (Figure 7).

### 3.4. Statistical Analysis

In Table 2, the results of the regression models are shown only for the gastrointestinal and genitourinary systems, where significant associations were found. No statistically significant results were observed for the other systems. However, a positive association was found between SNP rs2052129 and increased gastrointestinal symptoms. A similar trend might exist between SNP rs1049742 and more frequent genitourinary symptoms, although the *p*-value is quite significant.

### 3.5. DAO Deficiency Genetic Risk Score

**Figure 8:**
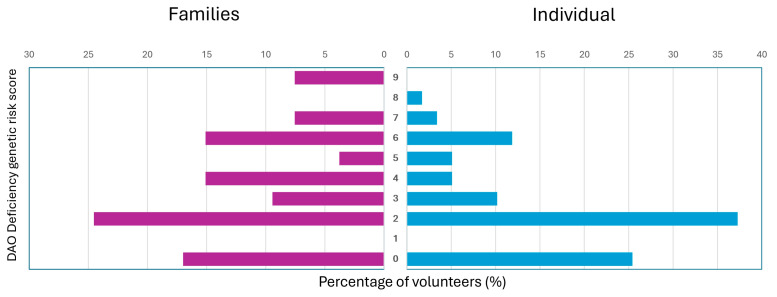
DAO deficiency genetic risk score.

Differences in the DAO deficiency genetic risk score were observed between individual volunteers and those grouped into families. Low scores (0 and 2) were more frequently found among individual volunteers, representing 25.42% and 37.29% of this group, respectively. In contrast, higher scores—specifically four (15.09%), six (15.09%), seven (7.55%), and nine (7.55%)—were more commonly observed in volunteers from family groups (Figure 8). Although no direct correlation was found between the DAO deficiency genetic risk score and the number of reported symptoms, elevated scores were consistently recorded in several family groups, particularly families 1, 2, 5, 7, 8, and 13.

### 3.6. Comparison Between Family Groups Versus Individual Volunteers

Differences in the frequencies of DAO SNPs between volunteers grouped into families and individual volunteers were evident. The altered allele was more frequent in family groups for SNPs rs2052129 (T = 0.311), rs10156191 (T = 0.330), and rs1049742 (T = 0.066); however, for rs1049793, the altered allele was more prevalent among individual volunteers (G = 0.356) (Table 3).

Based on the calculated p-values, most comparisons did not show statistically significant differences. However, exceptions were observed for the SNPs rs2052129 and rs10156191 in the sample of families when compared with the Latin American 2 reference population (Table 3).

Finally, the probability of having the different genotypes in the four analyzed DAO SNPs was determined under the Hardy–Weinberg equilibrium (Table 4). The probabilities of homozygosity for the altered allele were as follows: rs2052129 (5.8%), rs10156191 (5.4%), rs1049742 (0.2%), and rs1049793 (11.8%). Homozygosity for the wild-type allele exceeded 50% for the following SNPs: rs2052129 (57.6%), rs10156191 (59%), and rs1049742 (92.2%). In contrast, for rs1049793, the highest probability was observed for heterozygosity (45.1%).

All chi-squared tests yielded *p*-values greater than 0.05, suggesting that the populations are in Hardy–Weinberg equilibrium, as the deviations between observed and expected genotype frequencies were not statistically significant.

## 4. Discussion

About 1–3% of the population around the world has histamine intolerance (HIT), with gastrointestinal symptoms being the most prevalent [1]. A low-histamine diet is commonly recommended for individuals with HIT; however, symptoms often persist despite dietary adjustments. In recent years, several studies have focused on diamine oxidase (DAO), an enzyme directly involved in the metabolism of exogenous histamine from food. These studies suggest that DAO deficiency may be strongly related to elevated histamine levels, contributing to the development of HIT-related symptoms.

Although DAO deficiency is a multifactorial condition principally attributed to (1) genetic alterations (SNPs), (2) damage in the bowel mucosa due to inflammatory pathologies, and (3) inhibition of the enzyme produced by drugs [4], a recent study conducted by Okutan and collaborators (2023) [3] has shown that 74.5% of DAO deficiency cases are mainly attributed to genetic factors; furthermore, despite a total of 85 SNPs having been reported, only four stand out due to their well-established clinical relevance and frequency. Other potential functional loci, including variants affecting promoters or splicing, were not analyzed in this study due to limitations in sample size and assay availability and to ensure sufficient statistical power for genotype–phenotype correlations. Future studies should explore these additional loci to comprehensively understand the genetic basis of DAO deficiency.

In Spain, the SNP rs10156191 stands out for having a frequency of 53.1%, followed by the SNPs rs2052129, with 49%; rs1049793, with 48%; and finally, rs1049742, with 19% [3]. Similar percentages were reported by Duelo and collaborators (2024) [22]: rs1049793 (59%), rs10156191 (52%), rs2052129 (51%), and rs1049742 (18%). In Mexico, only one study has evaluated this topic to date, involving mothers of allergic children from northern Mexico (Torreón, Coahuila), where rs1049793 was reported as the most frequent (65%) [6].

In our study, considering all the volunteers, the frequencies were 39.29% for rs10156191, 38.39% for rs2052129, and 8.04% for rs1049742, which somehow correlates with the data reported by Okutan and collaborators (2023) [3], as far as order is concerned. In contrast, the frequency of rs1049793 in our population closely matches that reported in the Mexican study by Meza-Velázquez and collaborators (2017) [6] (62.5%) but not the frequencies observed in the Spanish population. Notably, Meza-Velázquez and collaborators found a high frequency of rs1049793 among patients with allergic rhinitis, which is consistent with the findings from our study, where allergy was the most prevalent symptom (*n* = 39/112; 34.82%).

Fifteen different DAO SNP combinations were identified among the volunteers, with genotype combination 2 being the most frequent (rs2052129, rs10156191, and rs1049742: wild-type homozygotes; rs1049793: heterozygote, *n* = 35). This finding is consistent with the study conducted by Okutan and collaborators (2023) [3], who reported fourteen different genotypic combinations in women with fibromyalgia. However, in their study, the most prevalent genotype consisted of wild-type homozygotes at all four DAO SNPs. Genotype combination 2 was expected to be the dominant type, as homozygosity for the wild-type allele exceeded 50% for rs2052129 (57.6%), rs10156191 (59%), and rs1049742 (92.2%). In contrast, rs1049793 showed the highest frequency of heterozygosity (45.1%). These findings are consistent with the data reported for Latin American populations but not with global data, highlighting the possibility that this specific genotype combination may be characteristic of this population.

To date, a minimum number of symptoms required for HIT diagnosis have not yet been established. In a 2019 study conducted by Schnedl and collaborators [10], 96.8% of HIT-diagnosed patients (*n* = 62) presented at least three symptoms in different organs, with an average of 11 out of 24 symptoms per patient. Similarly, Duelo and collaborators (2024) [22] reported that 99% of HIT patients exhibited more than three symptoms, with an average of eight symptoms per patient. Zhao and collaborators (2022) [15] suggested that HIT should be considered when symptoms affect two or more organs or systems, and symptom improvement can be observed following a low-histamine diet.

In the present study, 41.07% of the volunteers exhibited at least three symptoms across different systems and were thus considered potential HIT cases. The average number of symptoms reported was 5 out of the 39 evaluated, with 1 individual presenting as many as 16 symptoms. It is worth mentioning that none of the volunteers in this study had a formal HIT diagnosis. These findings highlight the current lack of standardized diagnostic criteria and underscore the difficulty in accurately determining the true incidence of HIT. Although current estimates suggest that 1–3% of the population may be affected by HIT, the actual prevalence is likely higher [7,14]. Improving awareness and implementing reliable diagnostic strategies are essential for obtaining more accurate epidemiological data.

The percentage of people around the world with DAO deficiency remains undefined; however, a study with a random population sample of 1051 healthy subjects reported 44% of cases [24]. Some reports described that heterozygotes (for the four described SNPs) prevailed [25]; however, no significant associations between the number of symptoms related to HIT, lower enzyme activity, and allele homozygosity or heterozygosity was found [16,26]. Moreover, Duelo and collaborators (2024) [22] reported a higher frequency of homozygous alleles in patients diagnosed with HIT. In fact, the absence of SNPs does not exclude the presence of HIT symptoms, which could be attributed to other factors. In our study, the ratio of wild-type homozygote, heterozygote, and mutated homozygote alleles within the four DAO SNPs were 62.95%, 31.25%, and 5.80%, respectively, with no mutated homozygote detected in the SNP rs1049742, as previously reported by other authors [25].

A total of 84.78% of the potential volunteers with HIT and 74.24% of the volunteers with less than three symptoms in different systems (considered healthy) presented at least one SNP; in both cases, genotype combination 2 was the most frequent (30.43% and 31.82%, respectively), followed by genotype combination 1. Okutan and collaborators (2023) [3] reported a prevalence of genetic DAO deficiency of 74.5%, while Duelo and collaborators (2024) [22] reported that 79% of individuals with symptoms of HIT harbored one or more SNP; however, no prevalence of any variant between patients and controls was evidenced. Despite more heterozygote SNPs being expected in the potential volunteers with HIT (genotype combination 10), interestingly, this genotype combination was more prevalent among healthy volunteers. In addition, 20 volunteers with different genotype combinations did not report any symptoms, highlighting the fact that DAO genotype combinations do not necessarily predict symptoms related to HIT, but the accumulation of multiple SNPs may indicate a relationship.

Although most of the volunteers carried one DAO SNP (37.5%), only 40.48% of them presented at least three or more symptoms in different systems associated with HIT; however, most of the volunteers carrying two DAO SNPs presented three or more symptoms (56.52%), and, finally, all the volunteers carrying four DAO SNPs presented symptoms. This data highlight the possible association between having four SNPs and the presence of HIT symptoms independently of the type of allele (heterozygote or mutated homozygote). However, it should be noted that the number of participants exhibiting this pattern (*n* = 9; 8.04%) was small, and further data are needed to support this observation.

In this study, symptoms corresponding to the nervous system were the most prevalent, followed by gastrointestinal symptoms and allergies; however, a study with 133 outpatients with HIT showed gastrointestinal manifestations in 92% of the patients, followed by cardiovascular, respiratory, and skin disorders [10]. It is worth mentioning that, in this study, a positive association was found between SNP rs2052129 and increased gastrointestinal symptoms.

SNPs are point mutations present in at least 1% of the population [17], which means that, since they are highly fixed mutations, they are likely to be inherited. The estimation of SNP inheritance may allow for the detection of the contribution of genetic factors to phenotypic variations. Although it is known that DAO SNPs can be inherited, no record exists of any study conducted on close relatives to date. In the present study, the probability of having a mutated allele was found to be 5.8% for rs2052129, 5.4% for rs10156191, 0.2% for rs1049742, and 11.8% for rs1049793, which matches with globally reported altered allele frequencies; however, inheriting one or more DAO SNPs was not indicative of inheriting any determined symptom related to HIT. Despite close family members sharing the same genotypic combinations, different HIT symptoms were reported.

As expected, the results varied between volunteers grouped into families or individual volunteers. In the case of volunteers grouped into families, higher DAO deficiency genetic risk scores were identified than in individual volunteers (scores of 9, 7, 6, and 4), which is explained by the frequency of inheriting DAO SNPs and its fixation within certain families (1, 2, 5, 7, 8, and 13). Wild-type homozygotes prevailed in individual volunteers, favoring lower DAO deficiency genetic risk scores (0, 2, and 3); however, no relationship between DAO deficiency genetic risk score and the number of symptoms was evidenced.

In future studies, serum DAO activity is desirable. However, it must be considered that it is a highly variable test that directly depends on the intake of certain food or drugs; the time of the day when the sample is taken; and, in the case of women, the menstrual cycle [3,4,27].

Finally, it should be considered that clinical manifestations associated with HIT could be improved through dietary interventions involving low-histamine intake or DAO supplementation, which may help to reduce intestinal dysbiosis by decreasing the abundance of certain histamine-secreting bacteria such as *Staphylococcus* and *Proteus* [2,5,28]. In this context, the identification of SNP carriers through genetic screening represents a promising strategy for the early diagnosis and improved characterization of patients presenting with unexplained symptoms such as migraine, gastrointestinal disturbances, or urticaria. This opens the possibility of implementing personalized interventions, including dietary recommendations, DAO supplementation, and microbiota modulation—tailored to the genetic background of each patient. Such an approach may enhance therapeutic effectiveness, improve quality of life, and reduce the socioeconomic burden of histamine-related disorders.

## 5. Conclusions

The prevalence of SNPs related to DAO deficiency in the Mexican population correlates with globally reported data; however, further analyses with volunteers distributed throughout the country would be desirable in order to provide more comprehensive data that reflect the nation’s genetic diversity. Although genetic predisposition was common, the presence of SNPs alone did not predict specific HIT symptoms. However, the accumulation of multiple SNPs is suggested to possibly increase the presence of HIT symptoms, regardless of the type of allele. These findings highlight the multifactorial nature of HIT and underscore the need for standardized diagnostic criteria.

## Figures and Tables

**Table 1:** General information on the diamine oxidase SNPs.

SNP	Amino Acid Change	Polymorphism		Context Sequence [VIC/FAM]
rs2052129	N/A	G/T	Transversion substitution	CGCCCTTCTCAACCTGACTAAGTGG**[G/T]**CGTTAGCACTGTCCGCCCACTGCAT
rs10156191	Thr16Met	C/T	Transition substitution	GTGGCTGCCATCCTGATGCTGCAGA**[C/T]**GGCCATGGCGGAGCCCTCCCCGGGG
rs1049742	Ser332Phe	C/T	Transition substitution	TTTGCCTTCCGGCTGCGCTCCTCCT**[C/T]**CGGGCTGCAGGTCCTGAACGTGCAC
rs1049793	His645Asp	C/G	Transversion substitution	CATCTACCACCAGAACGACCCCTGG**[C/G]**ACCCGCCCGTGGTCTTTGAGCAGTT

**Table 2:** Genotype–phenotype relationships.

Regressors	Gastrointestinal System		Genitourinary System	
	Odds Ratio	*p*-Value	Odds Ratio	*p*-Value
Age	1.01	0.43	1.00	0.84
Female	3.05	0.01	37.41	0.01
**rs2052129**	**15.16**	**0.03**	16.35	0.10
rs10156191	0.09	0.05	0.07	0.13
rs1049742	0.56	0.54	32.47	0.06
rs1049793	1.15	0.77	0.67	0.63

**Table 3:** DAO SNP frequencies.

SNP	rs2052129	rs10156191	rs1049742	rs1049793
**Global sample size**	580,272	452,454	501,300	114,592
Reference allele	G = 0.7651	C = 0.7299	C = 0.9288	C = 0.6805
Altered allele	T = 0.2349	T = 0.2701	T = 0.0712	G = 0.3195
**Latin American 1**	8318	6478	8370	790
Reference allele	G = 0.7795	C = 0.6874	C = 0.9397	C = 0.622
Altered allele	T = 0.2205	T = 0.3126	T = 0.0603	G = 0.378
**Latin American 2**	16,656	11,560	12,768	946
Reference allele	G = 0.8403	C = 0.8177	C = 0.9656	C = 0.614
Altered allele	T = 0.1597	T = 0.1824	T = 0.0344	G = 0.386
**This study/individual**	59	59	59	59
Reference allele	G = 0.822	C = 0.856	C = 0.983	C = 0.644
Altered allele	T = 0.178	T = 0.144	T = 0.017	G = 0.356
Comparisons with Latin American 1	*p* = 0.4324	*p* = 0.0054	*p* = 0.1628	*p* = 0.7365
Comparisons with Latin American 2	*p* = 0.7017	*p* = 0.4460	*p* = 0.4639	*p* = 0.6458
**This study/families**	53	53	53	53
Reference allele	G = 0.689	C = 0.670	C = 0.934	C = 0.670
Altered allele	T = 0.311	T = 0.330	T = 0.066	G = 0.330
Comparisons with Latin American 1	*p* = 0.1135	*p* = 0.7855	*p* = 0.8621	*p* = 0.4847
Comparisons with Latin American 2	*p* = 0.0027	*p* = 0.0056	*p* = 0.2086	*p* = 0.4144
**This study/total**	112	112	112	112
Reference allele	G = 0.759	C = 0.768	C = 0.960	C = 0.656
Altered allele	T = 0.241	T = 0.232	T = 0.040	G = 0.344
Comparisons with the global sample	*p* = 0.879	*p* = 0.364	*p* = 0.199	*p* = 0.578

Latin American 1: Latin American individuals with Afro-Caribbean ancestry; Latin American 2: Latin American individuals with mostly European and Native American Ancestry. From rs2052129 RefSNP Report—bSNP—NCBI, rs10156191 RefSNP Report—dbSNP—NCBI, rs1049742 RefSNP Report—dbSNP—NCBI, and rs1049793 RefSNP Report—dbSNP—NCBI [23].

**Table 4:** Genotype probability under the Hardy–Weinberg equilibrium.

SNP	rs2052129	rs10156191	rs1049742	rs1049793
**Global sample size**				
WT allele	GG = 0.5854, 58.5%	CC = 0.5327, 53.3%	CC = 0.8627, 86.3%	CC = 0.4631, 46.3%
Heterozygote allele	GT = 0.3594, 35.9%	CT = 0.3942, 39.4%	CT = 0.1322, 13.2%	CG = 0.4350, 43.5%
Mutated allele	TT = 0.0552, 5.5%	TT = 0.0729, 7.3%	TT = 0.0051, 0.5%	GG = 0.1021, 10.2%
Chi-squared value (*p*-value)	0.0078 (0.9295)	0.0024 (0.9610)	0.1115 (0.7385)	0.0014 (0.9707)
**Latin American 1**				
WT allele	GG = 0.6076, 60.8%	CC = 0.4725, 47.3%	CC = 0.8830, 88.3%	CC = 0.3868, 38.7%
Heterozygote allele	GT = 0.3437, 34.4%	CT = 0.4296, 43.0%	CT = 0.1133, 11.3%	CG = 0.4700, 47%
Mutated allele	TT = 0.0486, 4.9%	TT = 0.0977, 9.8%	TT = 0.0036, 0.4%	GG = 0.1429, 14.3%
Chi-squared value (*p*-value)	0.0002 (0.9899)	0.0001 (0.9904)	0.0046 (0.9458)	0.0010 (0.9744)
**Latin American 2**				
WT allele	GG = 0.7061, 70.6%	CC = 0.6686, 66.9%	CC = 0.9324, 93.2%	CC = 0.3770, 37.7%
Heterozygote allele	GT = 0.2685, 26.9%	CT = 0.2983, 29.8%	CT = 0.0664, 6.6%	CG = 0.4740, 47.4%
Mutated allele	TT = 0.0255, 2.6%	TT = 0.0333, 3.3%	TT = 0.0012, 0.1%	GG = 0.1490, 14.9%
Chi-squared value (*p*-value)	0.0000 (0.9965)	0.0004 (0.9850)	0.0006 (0.9800)	0.0005 (0.9814)
**This study/individual**				
WT allele	GG = 0.6756, 67.6%	CC = 0.7327, 73.3%	CC = 0.9663, 96.6%	CC = 0.4147, 41.5%
Heterozygote allele	GT = 0.2924, 29.2%	CT = 0.2465, 24.7%	CT = 0.0334, 3.3%	CG = 0.4586, 45.9%
Mutated allele	TT = 0.0317, 3.2%	TT = 0.0207, 2.1%	TT = 0.0003, 0.03%	GG = 0.1267, 12.7%
Chi-squared value (*p*-value)	0.0137 (0.9069)	0.0562 (0.8126)	0.0175 (0.8946)	0.0199 (0.8877)
**This study/families**				
WT allele	GG = 0.474, 47.4%	CC = 0.4489, 44.9%	CC = 0.8724, 87.2%	CC = 0.4489, 44.9%
Heterozygote allele	GT = 0.428, 42.8%	CT = 0.4422, 44.2%	CT = 0.1233, 12.3%	CG = 0.4422, 44.2%
Mutated allele	TT = 0.097, 9.7%	TT = 0.1089, 10.9%	TT = 0.0044, 0.4%	GG = 0.1089, 10.9%
Chi-squared value (*p*-value)	0.0077 (0.9302)	0.0190 (0.8905)	0.2650 (0.6067)	0.0190 (0.8905)
**This study/total**				
WT allele	GG = 0.5761, 57.6%	CC = 0.5898, 59.0%	CC = 0.9216, 92.2%	CC = 0.4303, 43.0%
Heterozygote allele	GT = 0.3660, 36.6%	CT = 0.3564, 35.6%	CT = 0.0768, 7.7%	CG = 0.4513, 45.1%
Mutated allele	TT = 0.0581, 5.8%	TT = 0.0538, 5.4%	TT = 0.0016, 0.2%	GG = 0.1183, 11.8%
Chi-squared value (*p*-value)	0.0247 (0.8752)	0.0004 (0.9849)	0.1963 (0.6578)	0.0096 (0.9218)

WT: Wild-type. Probability and percentage of each allele are shown.

## Data Availability

The original contributions presented in this study are included in the article/supplementary material. Further inquiries can be directed to the corresponding author(s).

## Supplementary Materials

The following supporting information can be downloaded at https://www.mdpi.com/article/10.3390/biomedicines13092280/s1, Table S1: DAO genotype combinations and symptoms related to HIT.
