# Supplementary material for: The Prevalence of Diamine Oxidase Polymorphisms and Their Association with Histamine Intolerance Symptomatology in the Mexican Population
Source: Biomedicines. 2025 Sep 17;13(9):2280. doi: 10.3390/biomedicines13092280 (PMC12467063; doi:10.3390/biomedicines13092280)
Supplement: Supplementary file 1 [file biomedicines-13-02280-s001.zip › biomedicines-3823059-supplementary.pdf]

Table S1. DAO genotype combinations and symptoms related to HIT.

Genotype combinations	# V	Family	Nerv	GI	Derm	Resp	GU	Cardiovasc	Insomnia	FMS	ALGY	ADHD
1	10	2	0	0	0	3	0	0	0	0	0	1
	19*	6	0	0	0	0	0	0	0	0	0	0
	28*	7	0	0	0	0	0	0	0	0	0	0
	34	9	0	1	0	0	0	0	0	0	0	0
	**35**	9	1	1	0	0	1	0	1	0	1	0
	**42**	11	4	4	2	0	1	2	1	1	1	0
	47	11	1	0	0	0	1	0	0	0	0	0
	**48**	12	1	2	0	0	0	0	1	0	0	0
	49	12	1	0	0	0	0	0	0	0	1	0
	61		0	1	0	0	0	0	1	0	0	0
	64		0	0	0	0	0	0	0	0	1	0
	65*		0	0	0	0	0	0	0	0	0	0
	66		0	0	0	1	0	0	0	0	0	0
	**69**		1	1	0	0	0	0	0	0	1	0
	73		0	0	1	0	0	0	0	0	0	0
	**78**		2	4	0	0	1	0	0	0	0	0
	81*		0	0	0	0	0	0	0	0	0	0
	92		0	0	0	0	0	0	0	0	1	0
	93*		0	0	0	0	0	0	0	0	0	0
	100		1	0	0	0	0	0	1	0	0	0
	**102**		2	2	3	0	0	0	0	0	1	0
	105*		0	0	0	0	0	0	0	0	0	0
	107		0	0	0	0	0	0	0	0	0	1
	**108**		2	4	0	0	0	0	1	0	0	0
2	**11**	3	2	1	2	0	2	0	1	0	1	0
	**12**	3	0	2	2	0	1	0	0	0	0	0
	**13**	4	1	1	1	0	0	0	0	0	1	0
	**14**	4	1	3	0	0	0	1	1	0	1	0
	16*	5	0	0	0	0	0	0	0	0	0	0
	**24**	7	1	0	0	0	0	0	1	0	1	0
	31	8	0	0	0	0	1	0	0	0	1	0
	36	10	3	1	0	0	0	0	0	0	0	0
	37	10	3	2	0	0	0	0	0	0	0	0
	38	10	0	0	0	0	0	0	0	0	1	0
	40	11	0	0	0	1	0	0	0	0	0	0
	**50**	12	1	1	0	0	1	0	0	0	0	0
	**52**	13	0	1	0	0	1	0	1	0	1	0
	**55**		0	3	1	0	0	0	1	0	1	0
	**56**		1	1	0	0	0	0	0	0	1	1
	**57**		1	1	0	0	2	0	0	0	0	0
	**62**		2	1	0	0	0	0	1	0	1	0
	63*		0	0	0	0	0	0	0	0	0	0
	68*		0	0	0	0	0	0	0	0	0	0
	70*		0	0	0	0	0	0	0	0	0	0
	71*		0	0	0	0	0	0	0	0	0	0
	72*		0	0	0	0	0	0	0	0	0	0
	74		0	1	0	0	0	0	1	0	0	0
	80		1	1	0	0	0	0	0	0	0	0

	82		3	3	0	0	0	0	0	0	0	0
	**83**		2	0	0	0	0	0	1	0	0	1
	85*		0	0	0	0	0	0	0	0	0	0
	86		0	0	0	3	0	0	0	0	1	0
	**88**		2	1	1	0	0	0	1	0	0	0
	**90**		0	1	0	1	0	0	1	0	1	0
	91		1	0	0	0	0	0	1	0	0	0
	95*		0	0	0	0	0	0	0	0	0	0
	103		1	1	0	0	0	0	0	0	0	0
	110		2	1	0	0	0	0	0	0	0	0
	112		0	0	0	0	1	0	0	0	0	0
3	**54**		2	2	1	1	1	0	0	0	0	0
4	39	10	0	0	0	1	0	0	0	0	1	0
	**58**		3	2	0	0	0	1	1	0	1	0
	**79**		3	4	0	0	1	0	1	0	1	0
	87		0	0	0	0	0	0	1	0	0	0
	109		1	0	0	0	0	0	0	0	0	0
	111*		0	0	0	0	0	0	0	0	0	0
5	23*	6	0	0	0	0	0	0	0	0	0	0
	**76**		3	4	0	0	1	0	1	0	1	0
6	20	6	0	0	0	4	0	0	0	0	1	0
	21*	6	0	0	0	0	0	0	0	0	0	0
	**44**	11	1	0	0	0	0	0	0	1	1	0
	45	11	1	0	0	0	0	0	0	0	0	0
7	**3**	1	1	1	0	0	0	0	1	0	0	0
	**8**	2	3	4	1	0	2	0	0	0	0	0
	**9**	2	1	3	0	0	1	0	1	0	0	1
	26	7	1	0	0	0	1	0	0	0	0	0
	27	7	0	0	0	1	0	0	0	0	1	0
	**41**	11	0	1	0	0	0	0	1	1	0	0
	**43**	11	1	4	2	0	0	0	0	0	0	0
	**46**	11	1	0	1	1	1	1	0	0	1	1
	**60**		1	1	0	0	1	0	0	0	0	0
	**75**		1	1	0	0	0	0	0	0	1	0
	**77**		7	2	1	2	1	0	1	1	0	0
8	5	1	0	2	0	0	0	0	0	0	0	0
	**96**		2	0	0	0	1	1	0	0	0	0
	99		1	0	0	0	0	0	0	1	0	0
9	**2**	1	2	3	0	3	0	0	1	0	1	0
	**29**	7	1	0	0	0	0	0	1	0	1	0
	59		2	0	0	0	0	0	0	0	0	0
10	15*	5	0	0	0	0	0	0	0	0	0	0
	17	5	1	2	0	0	0	0	0	0	0	0
	22	6	0	0	0	0	0	0	0	0	1	0
	**32**	8	1	1	0	0	0	0	0	0	1	0
	**51**	13	1	4	2	1	0	0	0	0	0	0
	**53**	13	2	1	0	0	0	0	1	0	0	1
	67		0	0	0	0	1	0	0	0	0	0
	84		0	0	0	0	0	0	0	0	1	0
	**94**		2	3	0	0	0	0	1	0	0	0
	97		3	0	0	0	0	0	0	0	1	0
	98		0	0	0	0	0	0	0	0	1	0
	104*		0	0	0	0	0	0	0	0	0	0

11	**18**	5	1	1	1	0	0	0	0	0	1	0
12	89*		0	0	0	0	0	0	0	0	0	0
13	1	1	0	3	0	0	0	0	0	0	0	0
	25	7	0	1	0	0	0	0	0	0	0	0
	**33**	8	1	0	0	0	2	0	0	0	1	0
	101		0	0	1	1	0	0	0	0	0	0
14	106		0	0	0	0	0	0	0	0	1	0
15	**4**	1	2	0	0	0	1	0	0	0	1	0
	**6**	1	1	0	0	1	0	0	0	0	0	1
	7	1	1	1	0	0	0	0	0	0	0	0
	30	8	0	0	0	1	1	0	0	0	0	0
Total #V			59	51	15	16	25	5	29	5	39	8

#V: Volunteer. *: Asymptomatic volunteers. Bold and underlined numbers: volunteers with at least three symptoms in different systems related to HIT. Members of the same family are shaded in each genotype. Colored numbers indicate the number of symptoms of each system. Systems: Nervous (Nerv): **1**: Anxiety; **2**: Chronic fatigue; **3**: Headache; **4**: Nervousness; **5**: Panic attacks; **6**: Depression; **7**: Generalized weakness; **8**: Dizziness; Gastrointestinal (GI): **1**: Bloating; **2**: Postprandial plenitude; **3**: Diarrhea; **4**: Abdominal pain; **5**: Constipation; **6**: Intestinal colic; **7**: Belching; **8**: Nausea; **9**: Vomiting; **10**: Flatulence; Dermatological (Derm): **1**: Pruritus; **2**: Redness; **3**: Eczema; **4**: Swollen eyelids; **5**: Red eyelids; Respiratory (Resp): **1**: Rhinorrhea; **2**: Rhinitis; **3**: Nasal congestion; **4**: Asthma/Bronchospasm; **5**: Frequent sneezing; **6**: Dyspnea; Genitourinary (GU): **1**: Menstrual cramping; **2**: Dysmenorrhea; **3**: Urinary symptoms; Cardiovascular (Cardiovasc): **1**: Hypotonia; **2**: Palpitations; **3**: Loss of consciousness; Insomnia; Fibromyalgia (FMS); Allergies (ALGY); ADHD (Attention Deficit Hyperactivity Disorder).
